# The Hydrodynamic Study of the Swimming Gliding: a Two-Dimensional Computational Fluid Dynamics (CFD) Analysis

**DOI:** 10.2478/v10078-011-0039-4

**Published:** 2011-10-04

**Authors:** Daniel A. Marinho, Tiago M. Barbosa, Abel I. Rouboa, António J. Silva

**Affiliations:** 1 University of Beira Interior. Department of Sport Sciences (UBI, Covilhã. Portugal).; 2 Research Centre in Sports, Health and Human Development (CIDESD, Portugal).; 3 Polytechnic Institute of Bragança. Department of Sport Sciences (IPB, Bragança. Portugal.; 4 University of Trás-os-Montes and Alto Douro. Department of Mechanical Engineering (UTAD, Vila Real, Portugal).; 5 University of Pennsylvania. Department of Mechanical Engineering and Applied Mechanics (UPENN, Pennsylvania. USA).; 6 University of Trás-os-Montes and Alto Douro. Department of Sports, Health and Exercise (UTAD, Vila Real. Portugal).

**Keywords:** tests and testing, computational fluid dynamics, technique, biomechanics, numerical simulations, swimming gliding

## Abstract

Nowadays the underwater gliding after the starts and the turns plays a major role in the overall swimming performance. Hence, minimizing hydrodynamic drag during the underwater phases should be a main aim during swimming. Indeed, there are several postures that swimmers can assume during the underwater gliding, although experimental results were not conclusive concerning the best body position to accomplish this aim. Therefore, the purpose of this study was to analyse the effect in hydrodynamic drag forces of using different body positions during gliding through computational fluid dynamics (CFD) methodology. For this purpose, two-dimensional models of the human body in steady flow conditions were studied. Two-dimensional virtual models had been created: (i) a prone position with the arms extended at the front of the body; (ii) a prone position with the arms placed alongside the trunk; (iii) a lateral position with the arms extended at the front and; (iv) a dorsal position with the arms extended at the front. The drag forces were computed between speeds of 1.6 m/s and 2 m/s in a two-dimensional Fluent^®^ analysis. The positions with the arms extended at the front presented lower drag values than the position with the arms aside the trunk. The lateral position was the one in which the drag was lower and seems to be the one that should be adopted during the gliding after starts and turns.

## Introduction

Competitive swimmers should have two aims to improve speed and thus enhance performance. They should: (i) maximize the propulsive forces produced by the propelling segments and; (ii) minimize the hydrodynamic drag resisting forward motion (Callaway et al., 2009; Marinho et al., 2009). Regarding the latter aim, a substantial energy is wasted to the water in order to overcome the resistance (Toussaint & Beek, 1992; Kolmogorov et al., 1997). Thus, expert swimmers seem to improve technique due to an increase in propulsive force, as well as, minimizing hydrodynamic drag (Seifert et al., 2007).

Efforts to minimize hydrodynamic drag should be carried-out during all swimming phases. However, decreasing drag during the gliding after starts and turns should be a main concern for swimmers and their coaches, especially nowadays, when the underwater gliding plays a major role to the overall swimming performance (Vilas-Boas et al., 2010). Thus, swimmers must adopt the most hydrodynamic position during gliding. Several studies (Vilas-Boas et al., 2000; Cossor & Mason, 2001) suggested that rather than the start technique used by the swimmer, it is his/her body position after immersion that mostly determines the success of the start. Indeed, there are several postures that the swimmers can assume during the underwater gliding, although experimental results were not conclusive concerning the best body position to perform this phase (Jiskoot & Clarys, 1975; Maglischo, 2003). Some swimmers prefer to glide in a lateral position and others in a prone one. In addition, during gliding swimmers can change their body posture. Moreover, in some techniques swimmers must change their limb positions. For instance, in breaststroke, the gliding is initially performed with the arms fully extended at the front. But then, swimmers perform a second gliding with the arms aside the trunk. It can be thought that these different postures might lead to differences in the intensity of the drag forces experienced by the swimmers.

Hydrodynamic drag consists of friction, pressure and wave drag. Friction, or viscous drag, is originated from fluid viscosity. It produces shear stresses in the boundary layer. Thus, the magnitude of friction drag depends on the wetted surface area of the body and the flow conditions within the boundary layer. Pressure, or form drag, is due to distortion of flow outside of the boundary layer. The flow over the swimmer’s body may separate at a certain point, depending on the shape, size and velocity of the swimmer. Behind the separation point, the flow reverses and may roll up into distinct eddies (vortices). As a result, a pressure differential arises between the front and the rear of the swimmer, resulting in pressure drag. When swimming near to the water surface, due to the interface between two fluids of different density, a third component of the total drag is due to wave drag. Kinetic energy from the swimmer is lost as it is changed into potential energy in the formations of waves (Toussaint & Truijens, 2005).

The drag force components produced by the swimmer gliding have been analysed applying different experimental methods (Clarys, 1979; Lyttle et al., 2000). However, as above mentioned, data obtained on those studies varied, which can represent some of the difficulties involved with experimental research (Bixler et al., 2007). Another different approach that can be used to analyse the effect of different postures during the gliding is the application of a numerical simulation method, such as computational fluid dynamics (CFD). Bixler et al. (2007) presented data supporting the accuracy and validity of this method to be used in swimming research. Since then, this methodology has been applied on regular basis to analyse the water flow around the human body and the forces involved during the swimmer’s displacement (Rouboa et al., 2006; Silva et al., 2008; Marinho et al., 2009). Therefore, the purpose of this study was to analyse the effect in hydrodynamic drag forces of using different body positions during gliding through computational fluid dynamics methodology.

## Material and Methods

### Digital model

Two-dimensional virtual models were created in CAD software to acquire the human body geometry. This geometry was based on the anatomical characteristics of a male national level swimmer’s group (n=15, 20.02±1.37 yrs, 1.87±0.21 m of height, 78.32±4.56 kg of body mass). Anthropometrical measures (height, arm span, arm length, leg lengths, hand length and width, foot length and width, and sitting height) of 15 male swimmers were assessed, assuming that the digital model represented average values of national level swimmers (participating in National Swimming Championships). Therefore, the digital model was created in CAD with 1.87 m height, a finger to toe length of 2.37 m (in the position with the arms extended at the front and with shoulders fully flexed) and, a vertex to toe length of 1.92 m (in the position with the arms along the trunk).

**Figure 1:**
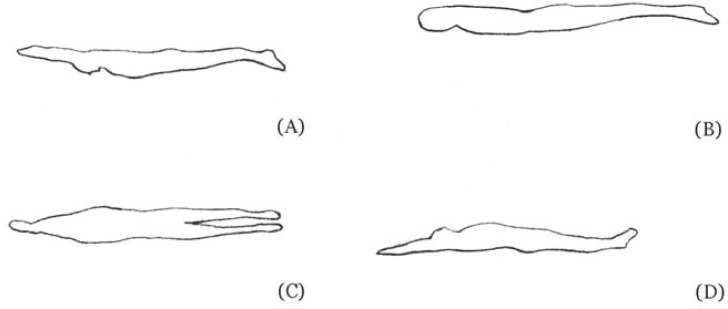
Swimmer’s model geometry with the body in the four different positions analysed in this study

Four digital models were developed to compare the swimmers’ posture gliding: (i) at the prone position, with the arms extended at the front of the body (Figure 1. Panel A); (ii) at the prone position with the arms placed alongside the trunk (Figure 1. Panel B); (iii) at the lateral position, with the arms extended at the front of the body (Figure 1. Panel C) and; (iv) at the dorsal position, with the arms extended at the front of the body (Figure 1. Panel D).

The models surfaces were then developed using Gambit^®^, a geometry modelling program of Fluent software (Fluent^®^, Inc. Hannover, USA). These surfaces were then meshed, using the same software, creating the grid which was imported into Fluent^®^ for analysis (Figure 1).

### Boundary conditions

The computational domain consisted of a two-dimensional grid, or mesh of cells, that simulate the fluid flow around the human body model.

The fluid mechanical properties, the flow characteristics along the outside grid boundaries and the mathematical relationship to account for the turbulence were considered. The standard k-epsilon turbulence model was considered and implemented in the Fluent^®^ software. This turbulence model was shown to be accurate with measured values in a previous research (Moreira et al., 2006).

The computational fluid dynamics analyses were done with the models in a horizontal position with an attack angle of 0º. The attack angle is defined as the angle between a horizontal plane and the foil cord (i.e. a line drawn from the head vertex to the ankle joint).

**Figure 2:**
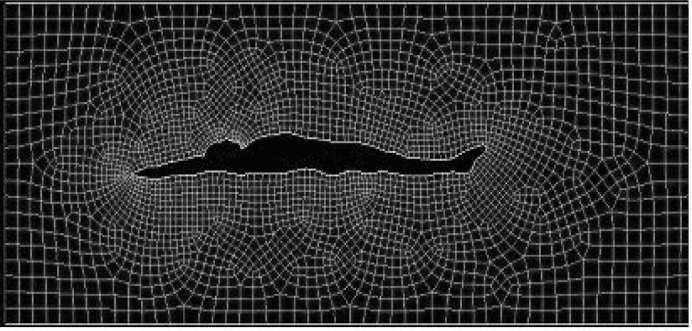
Computational fluid dynamics model geometry with the model in a dorsal position with the arms extended at the front

The boundary conditions of the computational fluid dynamics model were designed to represent the geometry and flow conditions of a part of a swimming pool lane. The water depth of the model was 1.80 m. The length of the computational domain was 8.0 m in the streamlined positions and 7.55 m in the position with the arms along the trunk, allowing in all situations the same flow conditions behind and in front of the model. In all positions, the distance from the swimmer’s hands (or head) to the front surface was 2.0 m and from the swimmer’s toe to the back surface was 3.63 m. The swimmer’s middle line (defined as a line passing through his/her centre of mass) was placed at a water depth of 0.90 m, an even distance from the top and the bottom surfaces (Figure 2).

The drag coefficients (Cd) and the drag force (Fd) were computed for speeds between 1.6 m/s and 2.0 m/s (with 0.1 m/s increments) in a two-dimensional Fluent^®^ steady flow analysis, according to equations 1 and 2:

(1) $$\mathrm{Fd}=0.5\cdot \rho \cdot \mathrm{SCd}\cdot v^{2}$$

(2) $$\mathrm{Cd}=\mathrm{Fd}/\left(0.5\cdot \rho \cdot S\cdot v^{2}\right)$$

where *Fd* is the drag force, *Cd* is the drag coefficient, *ρ* is the fluid density, *S* is the projection surface of the model and *v* is the flow speed.

### Data analysis

For each position and speed, drag coefficients and drag forces data were described and compared. The relationships between speed and drag coefficient, as well as speed and drag force were studied through a non-linear fitting mathematical model, through the application of various exponential equations. Added to the computing equations, the R^2^ (coefficient of determination) was also used as a measure of the models good-of-fit.

It was assumed that total drag force comes only from the friction and pressure drag components, as the model was placed 0.90 m underwater. Therefore, wave drag can be considered as negligent, and approximately close to null value.

## Results

Table 1 shows the drag coefficient values produced by the four body positions. The partial contribution of the friction and pressure drag components to total drag is also presented. The positions with the arms extended at the front showed drag values lower than the position with the arms aside the trunk. The lateral position presented the lowest values of total drag. Moreover, the ventral and the dorsal positions resulted in similar data.

Regarding each drag force component, pressure drag was the main factor responsible for total drag force, contributing to ∼92% and ∼85% of the total drag, at the position with the arms aside the trunk and at the positions with the arms extended at the front, respectively.

**Figure 3:**
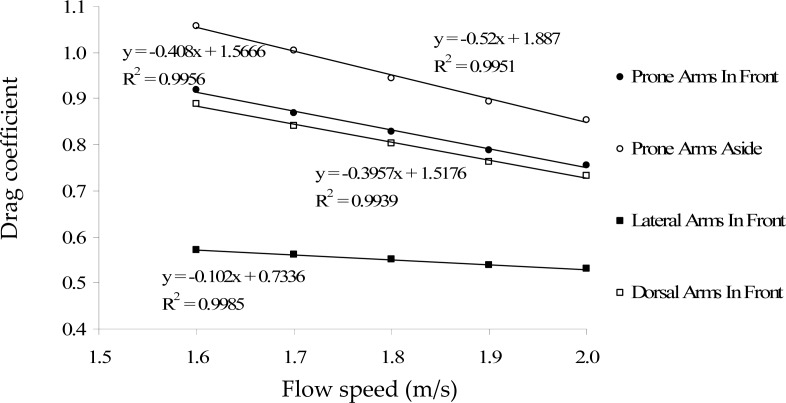
Relationship between the drag coefficient and the speed for the different gliding postures. The regression equations and the R^2^ values are also presented

**Figure 4:**
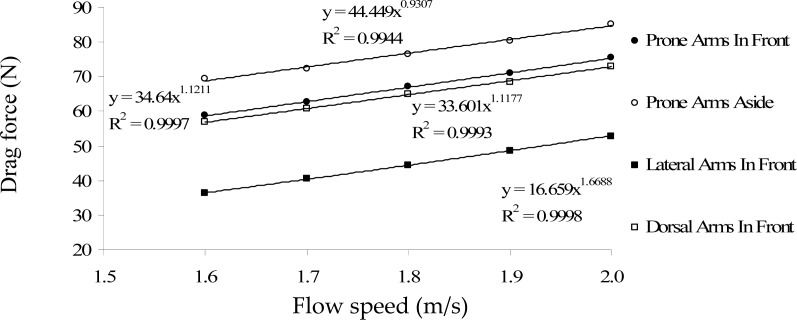
Relationship between the drag force and the speed for the different gliding postures. The equations and the R^2^ values are also presented

Figures 3 and 4 present the relationship between the drag coefficient and speed, as well as, the drag force and the speed, for the different gliding postures, respectively. For all studied positions, the drag coefficient values decreased with speed whereas the drag force values increased with speed flow.

## Discussion

The aim of this study was to analyse the effect in hydrodynamic drag forces when different body positions are applied during underwater gliding after starts and turns in swimming. Main data suggested that the positions with the arms fully extended at the front of the body presented lower drag values than the position with the arms aside the trunk. In addition, the lateral position (with the arms at the front) presented the lowest hydrodynamic drag during underwater gliding.

Nowadays, in biomechanical engineering the CFD technique is one of the best methods used for the analysis of fluid flow. The CFD is based on computer simulations, thus presents the advantage of testing several situations and allows to obtain the best or optimal result, without physical/experimental testing. The CFD was developed to be valid and accurate in a large scope of fluid environments, bodies and tasks, including sports, being scientifically assumed that the CFD has ecological validity even for swimming research (Bixler et al., 2007; Marinho et al., 2009). Bixler et al. (2007) compared the hydrodynamic drag between a digital CFD model of a male swimmer and a real mannequin based on the digital model. The authors (Bixler et al., 2007) found drag forces determined from the digital model using the CFD approach to be within 4% of the values experimentally determined for the mannequin. Hence, the CFD allows to examine changes in the swim stroke technique and how these changes might affect the swimmer’s performance. In the current work, we have analysed the hydrodynamic drag suffered by the swimmer at different underwater gliding body positions that are used in high-level events, attempting to help swimmers and their coaches to improve performance due to decreasing drag during this phase. This study was developed through a passive drag analysis, with the body in a fixed position, without any movements of the limbs, position assumed during the first part of the gliding after the start and after pushing-off from the wall during the turns (Konstantaki & Winter, 2007).

The four selected positions can be observed on a real swimming setting, after the starts and turns. For example, the gliding phase in front crawl, butterfly and the first gliding in breaststroke (at a prone position with the arms extended at the front), the second gliding in breaststroke (at a prone position with the arms aside the trunk), the gliding in backstroke (at a dorsal position with the arms extended at the front) and, in some techniques/phases during the gliding in front crawl (at a lateral position with the arms extended at the front).

Regarding drag coefficient values, one can note that there was an inverse relationship between this variable and the speed flow. The Cd decreased as speed increased. The inverse relationship between the drag coefficient and the speed found in the current study seems to correspond to what is reported with experimental settings for human bodies fully immersed (Jiskoot & Clarys, 1975; Lyttle et al., 1999). Jiskoot and Clarys (1975) found Cd values between 0.95 and 1.0, with a slope of the regression between the Cd and the speed of −0.17 (towing speeds ranging from 1.5 to 1.9 m/s). This data is similar to the one reported by Lyttle et al. (1999), −0.16, using speeds ranging from 1.6 to 3.1 m/s. In the current study, the values of the slope of the regression line varied between −0.52 and −0.10. It is interesting to notice that the most hydrodynamic positions presented the lowest slope values, and these values are similar to experimental data.

CFD data and experimental data presented quite similar Cd values (Clarys, 1979; Mollendorf et al., 2004). Clarys (1979) found Cd values between 0.58 and 1.04 for the human body in a passive towing situation. More recently, Mollendorf et al. (2004) also found in a passive towing approach Cd values between 0.83 and 0.90. Our data ranged from 0.53 to 1.06. However, if we only reported to the prone position (similar to the one used by Mollendorf et al., 2004) the CFD data are very similar to experimental data (Cd values ranged from 0.76 to 0.92).

Nevertheless, the current CFD data still presented some differences with the CFD results obtained by Bixler et al. (2007), using a three-dimensional model of the human body. These authors reported Cd values of 0.302, 0.300, 0.298 and 0.297 for speeds of 1.5, 1.75, 2.0 and 2.25 m/s, respectively. Probably, differences between two- and three-dimensional models lead to these differences. Additionally, one can add the different methodology to acquire the digital model. Bixler et al. (2007) carried-out laser scans of a male swimmer to obtain the boundaries of the human body; whereas in this study the model was designed in CAD.

Concerning the values of drag force, Miyashita and Tsunoda (1978) reported drag force values of 35.3 N for females; whereas Clarys (1979) presented values of 51.9 N for male national level swimmers, similar to the ones found in the current research. Lyttle et al. (1998), at a lower velocity studied (1.6m/s), and at a deeper towing position they studied (0.6m deep), also reported values for male swimmers within this range (58.1 N).

Our results are also similar to the ones found by Bixler et al. (2007) using a CFD approach. These authors found drag force values of 31.58, 42.74, 55.57 and 70.08 N for speeds of 1.5, 1.75, 2.0 and 2.25 m/s, respectively, with the human model at a prone position with the arms extended at the front. In this position we found drag force values from 58.7 to 75.4 N for speeds ranging between 1.6 and 2.0 m/s.

It was also found that the body position with the arms fully extended at the front presented lower Cd values than the body position with the arms aside the trunk. Furthermore, the lateral position presented much lower Cd values in comparison to others. The prone and the dorsal positions (both with the arms extended at the front) presented similar data.

The analysis of the passive drag was one of the first applications of biomechanics in swimming. The position with the arms extended at the front was the most studied position. This position is mostly accepted by the swimming technical and scientific communities as the most hydrodynamic one, being called the streamlined position (Guimarães & Hay, 1985; Goya et al., 2003). The values found in this study seemed to corroborate the assumption. The position with the arms fully extended at the front seems to smooth the anatomical shape especially at the head and shoulders. This could be explained by the “compressive” effect over the shoulders and chest width produced by the extended arms and may be one of the main determining factors associated to a reduced drag in these body postures. Thus, it seems possible to stress that, after breaststroke starts and turns, the first gliding position is biomechanically preferable compared to the second one. For this reason swimmers and coaches should allow it to prevail, and should also stress the need of body position control during its execution (Vilas-Boas, 2010). This aim should be accomplished to decrease drag and thus to improve gliding performance.

Regarding the positions with the arms extended at the front, the lateral position presented the lowest values of hydrodynamic drag. This finding is not quite simple to explain. Firstly, there are a scarce number of papers in the literature analysing passive drag at different body positions. Secondly, the use of a two-dimensional model can lead to some misinterpretation of the results and to not truly correspond to what happens in real swimming conditions.

Similar values between prone and dorsal positions, both with the arms extended at the front, seemed to be expected, since the hydrodynamic profiles of each position are similar. However, in the lateral position, the modelling of a two-dimensional model seemed to determine the obtained data. Counsilman (1955), just like Jiskoot and Clarys (1975), using experimental approaches, analysed prone and lateral towing positions. Nevertheless, their findings were contradictory for speeds lower than 1.8 m/s. For higher velocities, both studies reported higher values of hydrodynamic drag for the lateral position. In the current study the lateral position presented lower values of drag for all the studied speeds (1.6–2.0 m/s). The analysis of the hydrodynamic profile could explain this result. As one can note in Figure 1, the body in a lateral position lead to a better hydrodynamic body position, with the arms and hands at the front assuming a “drop of water” shape, situation that does not occur in the other two positions with the arms extended at the front. This “drop of water” shape seems to be the one that allows a better hydrodynamic profile, reducing pressure gradient around the body (Vogel, 1994). Indeed, the referred “drop of water” shape is more similar to other hydrodynamic shapes contributing to a smoother water flow around the human body (Massey, 1989). “Drop of water” shape keeps the boundary layer more time attached to the swimmers body surface. Thus, it delays the separation to a rear point of the body surface (Polidori et al., 2006).

The CFD also allowed to quantify the components of hydrodynamic drag and to evaluate the contribution of each one. The computed drag components showed that the pressure drag was dominant in all body positions. However, friction component cannot be negligible, since it represented 16%, 14%, and 8% in the lateral position, in the dorsal and prone positions, and in the prone position with the arms aside the trunk, respectively. The larger contribution of the friction component in the positions with the arms extended at the front may be related with the most hydrodynamic profile of this position during the gliding. This position allows the body to be more extended in the water, favouring the elongated position, which may allow both an increase of body length and slenderness, which reduces the pressure drag and may increase friction drag (Vogel, 1994).

Another situation could occur if the swimmer were at the water’s surface (Nicolas & Bideau, 2009). In the current study the swimmer model was placed underwater (0.90 m deep). In this sense, we did not consider the wave drag component since Lyttle et al. (1999) concluded that there was no significant wave drag contribution immersed at least 0.6 m. Therefore, during an underwater gliding more close to the surface the contribution of friction component would be reduced due possibly to the decrease of the wetted area and the generation of wave drag (Bixler et al., 2007). This concern seems to represent an interesting idea to develop in further CFD research.

The main limitations of this study include: (i) the analysis of a passive drag situation; the findings of this study must be read with caution, because we only analysed a passive drag situation. In the future, the development of this methodology must consider the body movements in the CFD domain, analysing, for instance, the second part of the gliding when the swimmer is kicking, allowing to study the total underwater phase (Konstantaki & Winter, 2007); (ii) the use of a two-dimensional model. The use of a simplified model of the human body does not allow a direct transfer of these data into a practical situation. In further research, this aim should be accomplished using three-dimensional models of the human body (Bixler et al., 2007).

As a conclusion, one can state that the positions with the arms fully extended at the front seem to substantially reduce the negative hydrodynamic effects of the human body morphology: a body with various pressure points due to the large changes in its shape. Thus, this posture performed at the lateral position should be the one adopted after the starts and turn phases of a competitive swimming event. For instance, considering the breaststroke turn, the first gliding, performed with the arms at the front, must be emphasized in relation to the second gliding, performed with the arms along the trunk.

## Figures and Tables

**Table 1:** Drag coefficient values and contribution of pressure and friction drag for the total drag to each speed and for the four different gliding postures

**Velocity (m/s)**	**Drag coefficient (Prone: arms aside the trunk)**			**Drag coefficient (Prone: arms extended at the front)**		

	Total drag	% Pressure drag	% Friction drag	Total drag	% Pressure drag	% Friction drag

1.6	1.06	92.23%	7.77%	0.92	86.13%	13.87%
1.7	1.01	92.24%	7.76%	0.87	86.15%	13.85%
1.8	0.95	92.29%	7.71%	0.83	86.16%	13.84%
1.9	0.89	92.30%	7.70%	0.79	86.23%	13.77%
2.0	0.85	92.34%	7.66%	0.76	86.24%	13.76%

	**(Dorsal: arms extended at the front)**			**(Lateral: arms extended at the front)**		

	Total drag	% Pressure drag	% Friction drag	Total drag	% Pressure drag	% Friction drag

1.6	0.89	85.91%	14.09%	0.57	83.87%	16.13%
1.7	0.84	86.01%	13.99%	0.56	83.88%	16.12%
1.8	0.80	86.07%	13.93%	0.55	83.91%	16.09%
1.9	0.76	86.12%	13.88%	0.54	83.98%	16.02%
2.0	0.73	86.14%	13.86%	0.53	84.05%	15.95%
